# Supplementary material for: Mapping the evolution of fertility support policies in China: A content and instrumental analysis
Source: PLoS One. 2025 Oct 9;20(10):e0332137. doi: 10.1371/journal.pone.0332137 (PMC12510515; doi:10.1371/journal.pone.0332137)
Supplement: S1 Appendix — (ZIP) [file pone.0332137.s001.zip › S1 Appendix. 226 original policy documents/181-国家计划生育委员会、国家民族事务委员会关于印发《关于加强少数民族人口与计划生育工作的意见》的通知(FBM-CLI-4-5055164).doc]

国家计划生育委员会、国家民族事务委员会关于印发《关于加强少数民族人口与计划生育工作的意见》的通知

**发布部门：** [国家计划生育委员会(已更名)](https://www.pkulaw.com/law/chl?Aggs.IssueDepartment=60314) [国家民族事务委员会](https://www.pkulaw.com/law/chl?Aggs.IssueDepartment=60303) [机构沿革](javascript:void(0))

**发布日期：**2000.11.13

**实施日期：**2000.11.13

**时效性：** [现行有效](https://www.pkulaw.com/law/chl?Aggs.TimelinessDic=01)

**效力级别：** [部门规范性文件](https://www.pkulaw.com/law/chl?Aggs.EffectivenessDic=XE0302)

**法规类别：** [人口计划管理](https://www.pkulaw.com/law/chl?Aggs.Category=09701) [少数民族事务综合](https://www.pkulaw.com/law/chl?Aggs.Category=00701)

国家计划生育委员会、国家民族事务委员会关于印发《关于加强少数民族人口与计划生育工作的意见》的通知

各省、自治区、直辖市、计划单列市计生委、民委，新疆生产建设兵团计生委、民委，中国人民解放军计划生育领导小组、武警总部计划生育领导小组办公室：
　　现将《关于加强少数民族人口与计划生育工作的意见》印发给你们，请结合实际情况认真贯彻落实。

二ΟΟΟ年十一月十三日

关于加强少数民族人口与计划生育工作的意见

　　控制人口增长，提高人口素质，是我国社会主义初级阶段长期面临的重大问题，也是关系到加快民族地区的经济发展，推进社会进步和提高人民生活水平，促进民族团结、社会稳定和各民族共同繁荣的重大问题。为了贯彻落实中央民族工作会议精神和《中共中央国务院[关于加强人口与计划生育工作稳定低生育水平的决定](https://www.pkulaw.com/chl/f827fcaffffe8d79bdfb.html)》（以下简称[《决定》](https://www.pkulaw.com/chl/f827fcaffffe8d79bdfb.html)），进一步做好少数民族和民族地区的计划生育工作，现提出以下意见：
　　**一、**认真总结经验，正确分析形势
　　九十年代以来，随着民族地区经济的发展，科技、教育、文化事业的进步和卫生条件的改善，少数民族的人口与计划生育工作取得了显著成绩：少数民族和民族地区人口自然增长过快的势头基本得到了控制，一些少数民族和民族地区已经实现了由高出生、低死亡、高增长到低出生、低死亡、低增长的人口再生产类型的转变；民族人口素质和育龄群众生殖健康水平有所提高；群众的生育观念发生明显的变化；综合治理人口问题的局面初步形成；计划生育“三为主”工作方针得到落实，基层基础工作得到加强，初步探索了一条民族地区开展计划生育工作的路子。
　　民族地区开展计划生育工作的基本经验是：
　　（一）各级党政领导、广大干部和少数民族群众深刻认识到，在少数民族和民族地区做好人口与计划生育工作，是加快民族地区发展，提高人民生活水平，促进民族繁荣进步，实现可持续发展的必由之路。实行计划生育已经成为各族群众的迫切要求。
　　（二）坚持两种生产一起抓，把计划生育工作纳入本地经济社会发展规划，统筹决策、综合治理，同发展区域经济、帮助群众脱贫致富、建设文明幸福家庭紧密结合，坚持在发展中解决人口问题。将计划生育与改善群众的生产和生活状况结合起来，得到了群众的理解和拥护。
　　（三）制定并执行符合民族地区实际、照顾少数民族特点的生育政策，保持政策的稳定性和连续性，做到既控制民族地区人口的过快增长，又保持少数民族人口的适度规模。
　　（四）尊重少数民族文化传统和风俗习惯，针对各民族特点开展工作，注意工作方法，增进民族团结，维护社会稳定。
　　（五）建立健全计划生育管理和服务网络，在计划生育工作中推进优生优育，开展生殖保健服务，提高出生人口素质。
　　（六）加强计划生育干部队伍建设，建立一支吃苦耐劳、无私奉献的各民族计划生育工作队伍。
　　但是，从总体上看，少数民族和民族地区的计划生育工作发展还很不平衡。目前，大部分少数民族和民族地区的人口再生产类型仍处于转变过程中，一些地区还没有摆脱“越穷越生，越生越穷”的恶性循环；早婚、早育的情况较为普遍，已婚育龄妇女的综合避孕节育率和计划生育率较低，文化素质偏低，文盲、半文盲人口比重较高，出生人口缺陷发生率较高，婴儿死亡率、孕产妇死亡率偏高；计划生育工作发展不平衡，不少地区基层基础工作薄弱，经费投入严重不足。

　　**二、**今后十年少数民族和民族地区人口与计划生育工作的目标和主要任务
　　今后十年，是我国实施西部大开发战略的重要历史时期，也是稳定低生育水平的关键时期。在西部大开发的诸因素中，人口因素是重要的决定性因素之一。能否有效地控制人口过快增长，提高民族人口素质，是实施西部大开发战略、实现各民族共同繁荣的重要保证。做好民族地区的人口与计划生育工作，也有利于维护民族团结、国家统一和边疆稳定。各级党委、政府务必认清形势，抓住西部大开发的有利时机，把人口与计划生育工作纳入本地区的发展战略，制定人口与计划生育工作同西部大开发战略紧密结合的政策和措施，正视存在的困难和问题，采取切实有效的措施，有目标、分阶段地推进民族地区人口与计划生育工作稳定健康地向前发展。
　　到2010年，民族地区计划生育工作的目标是：育龄群众的婚育观念发生显著变化，基本实现按政策生育，人口总量得到有效控制，出生人口素质明显提高；育龄群众享有基本的生殖保健服务，逐步开展避孕方法的“知情选择”，努力实现孕前型管理与服务，综合避孕率稳定在80%以上，计划生育手术并发症发生率控制在1‰以下；出生婴儿性别比保持基本正常；基本实现工作思路和工作方法的“两个转变”， 形成具有民族地区特色的计划生育管理机制、服务体系和综合治理人口问题的局面。
　　为实现上述目标，必须坚持以下原则：一是把人口与计划生育工作纳入西部大开发战略，统筹决策，综合治理。二是坚持和稳定现行的少数民族生育政策。三是坚持分类指导，逐步推进。根据不同民族、不同经济水平和不同的工作基础，确定不同的目标。四是以人的全面发展为中心，维护群众合法权益。坚持行政管理与群众工作相结合，提高依法行政水平，依靠科技进步，为群众提供优质服务。五是坚持民族团结，执行民族政策，尊重民族风俗习惯，发挥宗教界和民族代表人士在计划生育工作中的特殊作用。
　　今后一个时期少数民族地区计划生育工作的主要任务是：
　　（一）增强责任意识，进一步控制民族地区人口过快增长，实现人口、经济、社会、资源、环境的协调发展和可持续发展。
　　未来一个时期，少数民族地区仍将面临很大的人口压力，与民族地区的经济发展、社会进步、资源、环境的承载能力很不适应。各民族地区要牢固树立人口意识和忧患意识，结合实际，确定本地区适度控制人口增长的目标。要大力提倡晚婚晚育，实行有间隔地生育，努力降低不符合政策生育的多孩率，提高计划生育率，把人口增长率控制在适当的水平。要充分考虑到各民族、各地区之间的差异性，对于人口增长已降低到较低水平的民族和地区，要稳定低生育水平，注意研究解决低生育水平下的人口问题；对于人口增长过快，计划生育率较低的民族和地区，要采取有效措施控制人口过快增长；对于生存条件恶劣的地区，要特别注意将计划生育工作与妇幼保健工作结合起来，降低孕产妇死亡率和婴儿死亡率，提高少数民族群众的健康水平，促进民族发展和民族繁荣。
　　（二）坚持以人的全面发展为中心，积极推进以技术服务为重点的优质服务，努力提高出生人口素质。
　　从满足少数民族地区群众的需求出发，实施避孕节育措施的“知情选择”、出生缺陷干预和妇女病的普查防治三大工程，根据育龄群众的不同需求，努力为群众提供避孕节育全程服务和生殖保健服务。因地制宜地推广适合民族特点和民族文化传统、群众容易掌握的避孕节育措施，积极稳妥地推进避孕方法的知情选择，提高避孕措施的有效性和安全性。要严格技术服务规范和标准，提高技术水平和服务质量，努力降低手术并发症发生率，努力避免避孕节育措施的严重不良反应和手术事故的发生。加强技术指导和咨询服务，提高群众的自我保健意识和能力，改变有利于身心健康的生活方式。 大力开展优生优育、婚前检查、产前诊断、遗传咨询和新生儿疾病筛查等预防性技术服务，不断提高少数民族和民族地区的出生人口素质。
　　（三）切实加强计划生育技术服务网络建设，不断满足少数民族群众对计划生育技术服务的需求。
　　要根据民族地区的实际，合理配置卫生资源，采取固定站与流动服务相结合的方式，改善乡级计划生育服务机构的技术服务条件，使之成为中心服务站（所）；有条件的乡可以单独建计划生育服务站（所）；不具备条件的，可与乡级卫生院合建，但要保证有相对独立的内设机构以及相应的房屋、设备、人员，并接受计划生育行政部门的业务指导；没有乡卫生院或乡卫生院技术力量十分薄弱的地方，可以建立小而精的计划生育技术服务机构。要配备必要的技术服务、生殖保健医疗设备和基本宣传设备，以利于计划生育工作的开展。具体做法由各级党委、政府予以协调和确定。在乡镇机构改革中不宜采取行政手段将计划生育服务机构并入其他机构。
　　（四）深入做好计划生育宣传教育工作，积极建设社会主义生育文化，引导群众树立科学、文明、进步的婚育观念。
　　要利用各种传媒，采取多种形式，有针对性地开展人口与计划生育宣传教育。深入开展“婚育新风进万家”和文化、科技、卫生“三下乡”活动，结合农村普及义务教育和扫盲活动，开展国情、国策和法律法规教育，大力普及计划生育、优生优育、生殖保健科普知识，引导群众崇尚科学，破除迷信，提高社会主义精神文明水平。发挥县、乡（镇）、村人口学校的作用，改进宣传内容、宣传方法，根据民族特点、风俗习惯，开展有针对性的面对面的宣传教育和有本民族特色的宣传活动，把耐心细致的思想工作同解决群众生产、生活、生育中的实际困难结合起来。努力开发、制作适合民族文化特点的宣传品；80%的县级电台、电视台每周有固定的多语种人口与计划生育节目；85%以上的育龄群众能够了解三种以上的避孕节育方法。发挥宗教界和民族代表人士在计划生育工作中的特殊作用。加强基层计划生育协会的建设，引导群众自我教育，自我管理，自我服务。
　　（五）广泛开展计划生育“三结合”和“兴边富民”行动。
　　把计划生育工作与发展民族地区经济、帮助少数民族群众脱贫致富、建设文明幸福家庭结合起来。要通过制定有利于计划生育的相关社会经济政策，采取小额贷款、项目优先、科技扶持等优先、优惠、优待政策，帮助计划生育家庭改善基本生产、生活条件，增强自我发展能力，率先脱贫致富。积极开展“救助贫困母亲”的幸福工程，大力推进“兴边富民”行动，根据实际情况，采取多种形式，通过多种渠道，逐步建立计划生育的利益导向机制和有利于计划生育的社会保障制度，特别是建立独生子女户和两女户养老保障制度，解除群众实行计划生育的后顾之忧。
　　（六）积极改革创新，认真落实计划生育“三为主”工作方针，加快实现工作思路和工作方法“两个转变”的步伐。
　　要根据民族地区的特点，在服务方式、资源配置和队伍建设方面进行大胆探索和改革。大力加强基层基础工作，健全村、组工作网络，扎扎实实开展创建计划生育合格村活动。坚持依法行政，推进村民自治，实行计划生育政务公开和村务公开，自觉接受群众监督。改革和完善人口与计划生育目标管理责任制，发挥考核的正确导向作用，以群众是否满意作为衡量工作的标准；考核指标的设置要体现分类指导，体现工作的发展方向。要通过考核把基层的注意力和工作重点引导到为群众提供优质服务、提高工作水平上来。进一步贯彻计划生育工作既要抓紧又要抓好的指导思想，把管理与服务有机结合起来，寓管理于服务之中。

　　**三、**切实加强对少数民族和民族地区人口与计划生育工作的领导
　　（一）各级党政领导要站在“三个代表”的高度，站在可持续发展的高度，从促进少数民族和民族地区团结、稳定和繁荣的高度出发，把人口问题纳入当地经济社会发展的总体规划，使计划生育工作与发展民族经济、消除贫困、保护生态环境、发展文化教育、提高妇女地位、增进人民健康等工作结合起来。自觉坚持党政一把手亲自抓、负总责，组织协调各方面力量齐抓共管，切实做到责任到位，措施到位，投入到位。要教育党员、干部模范执行计划生育政策，做自觉实行计划生育的表率。基层干部和计划生育工作人员要努力提高自身素质，增强为人民服务的意识和法制观念，切实转变工作作风，维护群众合法权益，关心群众生活，积极化解矛盾，维护社会稳定和民族团结。
　　（二）加大对计划生育工作的投入。在西部大开发战略中，要加大对计划生育工作的投入，尤其是要加大对少数民族地区的倾斜力度。要按照中央的要求，把计划生育经费纳入财政预算，切实予以保证。要按照中央[《决定》](https://www.pkulaw.com/chl/f827fcaffffe8d79bdfb.html)的要求，逐步提高计划生育经费投入的总体水平，计划生育事业费的增长幅度要高于财政收入的增长幅度。对少数民族财政困难地区的计划生育事业费的投入的增长要高于同级财政对计划生育事业费投入的增长幅度。社会抚养费、乡统筹费纳入财政预算后，财政要相应增加计划生育经费的投入。要广开渠道筹集计划生育经费，鼓励对口支援和民间捐助、社会捐助和国际捐助。在有条件的地方建立人口与计划生育公益基金等。
　　国家要进一步加大对计划生育基本建设项目和事业费分配的倾斜力度。“十五”期间，继续完成向国家重点扶持的贫困县（包括少数民族贫困县）配备流动服务车的工作，加强计划生育技术服务设施建设。要积极争取中央财政通过转移支付的方式逐步增加对少数民族地区的照顾，适当增加补助少数民族地区经费的比例。选择少数民族贫困地区，开展农村计划生育养老保障试点工作。
　　（三）高度重视计划生育干部队伍建设，建立一支思想好、作风正、懂业务、会管理、善于做群众工作的管理队伍、技术队伍和群众工作队伍。要加强各民族干部之间的团结，树立汉族离不开少数民族，少数民族离不开汉族，各少数民族之间相互离不开的思想。注意选拔德才兼备的同志充实到领导岗位，重视选拔、使用年轻干部，特别要注意培养、使用少数民族干部和妇女干部。要重点加强行政管理人员和专业技术人员的配备和培训，不断提高他们的管理水平和业务素质，并妥善解决计划生育技术服务人员的职称问题。力争在2005年以前对现有的管理、技术服务人员普遍轮训一次。逐步在乡、村两级形成以专业技术人员为主体，一专多能、适应优质服务需要的年轻化的各民族计划生育工作队伍。要关心、爱护计划生育干部，为他们创造工作条件，落实报酬，计划生育干部的岗位津贴要优先得到保障，以使他们安心工作。要改革干部人事制度，在干部队伍建设中引进竞争机制，实行公开招聘，竞争上岗。
　　（四）加强对口支援。东部各省（市）要同民族地区结成帮扶对子，开展对口支援，在经验交流、技术支持、设备援助、人员培训等方面支持民族地区。东部地区要选派有经验的技术人员通过代培、挂职等形式为少数民族地区培养管理人员和技术服务人员；少数民族地区要选派技术人员到东部地区学习、实习，提高科学管理和技术服务技能。国家计生委将出台帮扶方案，东部各省要拿出具体的帮扶计划，帮扶对子落实到县。对帮扶的效果要进行检查和评估。
　　做好少数民族和民族地区的人口与计划生育工作，任重道远，我们一定要从战略的高度，从各民族人民的根本利益出发，保持清醒头脑，坚持科学态度，把少数民族和民族地区的计划生育工作提高到一个新的水平。

© 北大法宝：（[www.pkulaw.com](https://www.pkulaw.com/)) 专业提供法律信息、法学知识和法律软件领域各类解决方案。北大法宝为您提供丰富的参考资料，正式引用法规条文时请与标准文本核对。
欢迎查看所有[产品和服务](https://www.pkulaw.com/helps/2.html)。法宝快讯：[如何快速找到您需要的检索结果？](https://www.pkulaw.com/helps/70.html)[法宝](https://www.pkulaw.com/helps/69.html)V6有何新特色？

扫描二维码阅读原文

**原文链接：**<https://www.pkulaw.com/chl/cf34754a2974b587bdfb.html>
